# The changes of CD4+CD25+/CD4+ proportion in spleen of tumor-bearing BALB/c mice

**DOI:** 10.1186/1479-5876-3-5

**Published:** 2005-01-28

**Authors:** Ji-Yan Liu, Xiao-Shi Zhang, Ya Ding, Rui-Qing Peng, Xia Cheng, Nian-Hua Zhang, Jian-Chuan Xia, Yi-Xin Zeng

**Affiliations:** 1 State Key Laboratory of Oncology in South China, Cancer Center, Sun Yat-sen University, Guangzhou, China; 2 Department of Biotherapy, Cancer Center, Sun Yat-sen University, Guangzhou, China

**Keywords:** CD4+CD25+ T_R _cells, mouse tumor model, splenic lymphocytes

## Abstract

CD4+CD25+ regulatory T lymphocytes (T_R_) constitute 5–10% of peripheral CD4+ T cells in naive mice and humans, and play an important role in controlling immune responses. Accumulating evidences show that T_R _cells are involved in some physiological processes and pathologic conditions such as autoimmune diseases, transplantation tolerance and cancer, and might be a promising therapeutic target for these diseases.

To evaluate the change of CD4+CD25+ T_R _cells in mouse tumor models, CD4+CD25+ subset in peripheral blood and spleen lymphocytes from normal or C26 colon-carcinoma-bearing BABL/c mice were analyzed by flow cytometry using double staining with CD4 and CD25 antibodies.

The proportion of CD4+CD25+/CD4+ in spleen lymphocytes was found to be higher than that in peripheral blood lymphocytes in normal mice. No difference was observed in the proportion in peripheral blood lymphocytes between tumor bearing mice and normal mice, while there was a significant increase in the proportion in spleen lymphocytes in tumor bearing mice as compared with normal mice. Moreover, the proportion increased in accordance with the increase in the tumor sizes. The increase in the proportion was due to the decrease in CD4+ in lymphocytes, which is resulted from decreased CD4+CD25- subset in lymphocytes. Our observation suggests the CD4+CD25+/CD4+ proportion in spleen lymphocytes might be a sensitive index to evaluate the T_R _in tumor mouse models, and our results provide some information on strategies of antitumor immunotherapy targeting CD4+CD25+ regulatory T lymphocytes.

## Background

Early in 1970s, the concept of suppressor T cells was developed and it was envisioned that this subset of lymphocytes was responsible for the active control, and ultimately the termination, of immune responses [1]. But the characters of this subset had not been well studied mainly because its distinct phenotype was not identified. In 1990s, Sakaguchi et al found that a subset of CD4+ lymphocytes in peripheral blood of normal mice expressed the IL-2R-α (CD25) and it down-regulated the immune response to self and non-self antigens [2]. Soon the CD4+CD25+ lymphocytes were verified as one group of suppressor T cell and termed as thymic derived "naturally occurring" regulatory T cells (T_R_). T_R _represents a minor (5–10%) component of peripheral CD4+ T cells but plays an important role in controlling immune responses [3]. Accumulating evidences show that T_R _cells possess potent suppressive activity both in vivo and in vitro and are involved in autoimmune diseases, transplantation tolerance and tumor immunity [2-5]. The transfer of CD4+CD25- cells into nude mice resulted in autoimmune diseases; reconstitution of CD4+CD25+ cells after transfer of CD4+CD25- cells prevented the development of autoimmunity [2]. Similarly, depletion of these cells induced gastritis and late-onset diabetes [6], impaired development or dysfunction of these cells increased susceptibility to experimental autoimmune encephalomyelitis [7], multiple sclerosis [8] and other autoimmune diseases [9,10]. Conversely, an increased percentage of CD4+CD25+ T_R _cells in total CD4+ T cells was found in peripheral blood of cancer patients [11-14] and depletion of CD25+ cells alone or combination with other strategies might cause tumor regression [4,15,16]. All these studies indicated the importance of T_R _cells in controlling immune response. The mechanism of how the T_R _cells control immune response is still unclear. Previous studies show that activated T_R _cells strongly inhibit proliferative responses of CD4+ or CD8+ T cells in vitro [17,18], moreover, it down-regulates co-stimulatory molecules on dendritic cells (DC) [19], inhibit the maturation and antigen-presenting function of DC [20], and suppress activated and matured DC driven responses [21]. The important role of T_R _cells in immunoregulation makes it be recognized as an attractive therapeutic target for immune-related diseases.

In our animal experiments of antitumor immunotherapy that targeting CD4+CD25+ T_R _cells, to our surprise, we did not find an increase of CD4+CD25+/CD4+ in peripheral blood of tumor bearing BALB/c or C57BL/6 mice, this is not in accordance with the increase of the proportion in cancer patients as reported by Wolf et al [11]. In order to find a way to evaluate the CD4+CD25+ T_R _cells in tumor-bearing mice, we analyzed CD4+CD25+ subset in peripheral blood and spleen lymphocytes from normal or C26 colon-carcinoma-bearing mice by flow cytometry.

## Methods

### Mice and tumor model

6 to 8 weeks BALB/c mice were purchased from the Laboratory Animal Center of Sun Yet-sen University. Mouse C26 colon carcinoma cell line was a gift from Prof. Li-Jian Xian (Cancer Center, Sun Yet-sen University). The C26 Cells were cultured in RPMI 1640 medium (Gibco Invitorogen Corporation) supplemented with 10% fetal calf serum (FCS; Gibco Invitorogen Corporation, Carlsbad, CA), 100 U/ml of penicillin G and 100 μg/ml of streptomycin, and the medium was renewed every 2 to 3 days. After growing to confluency, the cells were detached with trypsin-EDTA, resuspended in serum-free RPMI 1640 medium and inoculated subcutaneously at right axilla with 1 × 10^5 ^to 1 × 10^7 ^live tumor cells per mouse.

### Reagents

PE-conjugated anti-mouse CD4, Cychrome-conjugated anti-mouse CD25 antibodies were purchased from eBioscience. Red blood cell lysis buffer is composed of 0.155 M ammonium chloride, 0.01 M potassium bicarbonate, and 0.1 mM EDTA. Fixation solution contains 1% paraformaldehyde in PBS.

### Samples preparation and flow cytometry

Mouse peripheral blood was collected from orbital plexus and anticoagulated with 20 U/ml sodium heparin. Single-cell suspensions of splenocytes were prepared by grinding the spleen with the plunger of a disposable syringe, passing the ground spleen through nylon mesh, and suspending the cells in PBS. Mouse peripheral blood or spleen single-cell suspensions were stained with PE-conjugated anti-mouse CD4 and Cychrome-conjugated anti-mouse CD25 antibodies at 4°C for 30 minutes. Then, erythrocytes were lysed by red blood cell lysis buffer. After wash with PBS, the samples were fixed with fixation solution and analyzed on a FACScalibur™ flow cytometer (BD Biosciences) with CELLQuest™ software.

### Statistical Analysis

The data are summarized as the mean ± standard error. Statistical analysis was performed using the Student *t *test, statistical significance was accepted at the *P *< 0.05 level.

## Results

### CD4+CD25+/CD4+ in peripheral blood and spleens from normal BALB/c mice

**Figure 1:**
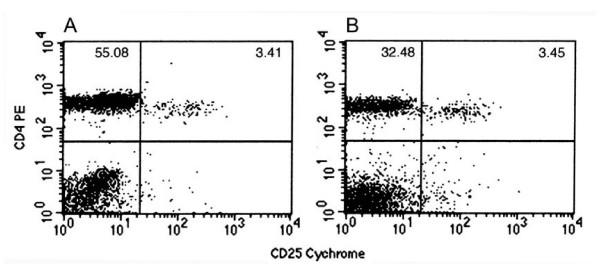
The proportions of CD4+CD25+ subset in peripheral blood and spleen lymphocytes from normal BALB/c mice. Mouse peripheral blood (A) or spleen single-cell suspensions (B) were collected or prepared, and stained with PE-conjugated anti-mouse CD4 and Cychrome-conjugated anti-mouse CD25 antibodies, after the lysis of erythrocytes, the samples were analyzed by flow cytometry.

To evaluate the normal proportion of CD4+CD25+/CD4+ in mice, 6 to 8 weeks normal BALB/c mice (n = 10) were sacrificed to test the proportion in spleen and peripheral blood lymphocytes by flow cytometry using anti-mouse CD4 and CD25 antibodies. The total CD4+ lymphocytes, CD4+CD25+ subset, and CD4+CD25+/CD4+ in spleen or peripheral blood lymphocytes were shown in Table 1. In normal mice, the CD4+CD25+ T_R _cells appear in spleen or peripheral blood in a relative stable percentage manner. The proportion of CD4+CD25+/CD4+ in peripheral blood was 6.19 ± 0.86%, which is in accordance with the results reported by others [3]. Otherwise, the CD4+CD25+/CD4+ proportion in spleen was higher than that in peripheral blood (10.23 ± 1.88% vs 6.19 ± 0.86%, *P *< 0.001). And, the higher level of the proportion in spleen is due to a lower level of the total CD4+ lymphocytes (CD4+CD25+ plus CD4+CD25-) in spleen than that in peripheral blood (37.06 ± 5.76 vs 56.80 ± 6.38, *P *< 0.001). The representable figures of peripheral blood and spleen lymphocytes double stained with CD4 and CD25 antibodies were shown in Figure 1.

**Table 1:** The percentages of CD4+CD25+ and CD4+, and the proportions of CD4+CD25+/CD4+ in peripheral blood and spleen lymphocytes from normal BALB/c mice.

	CD4+	CD4+CD25+	CD4+CD25+/CD4+	total lymphocyte
peripheral blood (n = 10)	56.80 ± 6.38	3.50 ± 0.45	6.19 ± 0.86	6.73 ± 0.84 (10^9^/L)
spleen (n = 10)	37.06 ± 5.76	3.79 ± 0.93	10.23 ± 1.88	1.54 ± 0.23 (× 10^8^)
*P *value	<0.001	0.38	<0.001	-

### CD4+CD25+/CD4+ in peripheral blood and spleens from C26 tumor-bearing BALB/c mice

**Figure 2:**
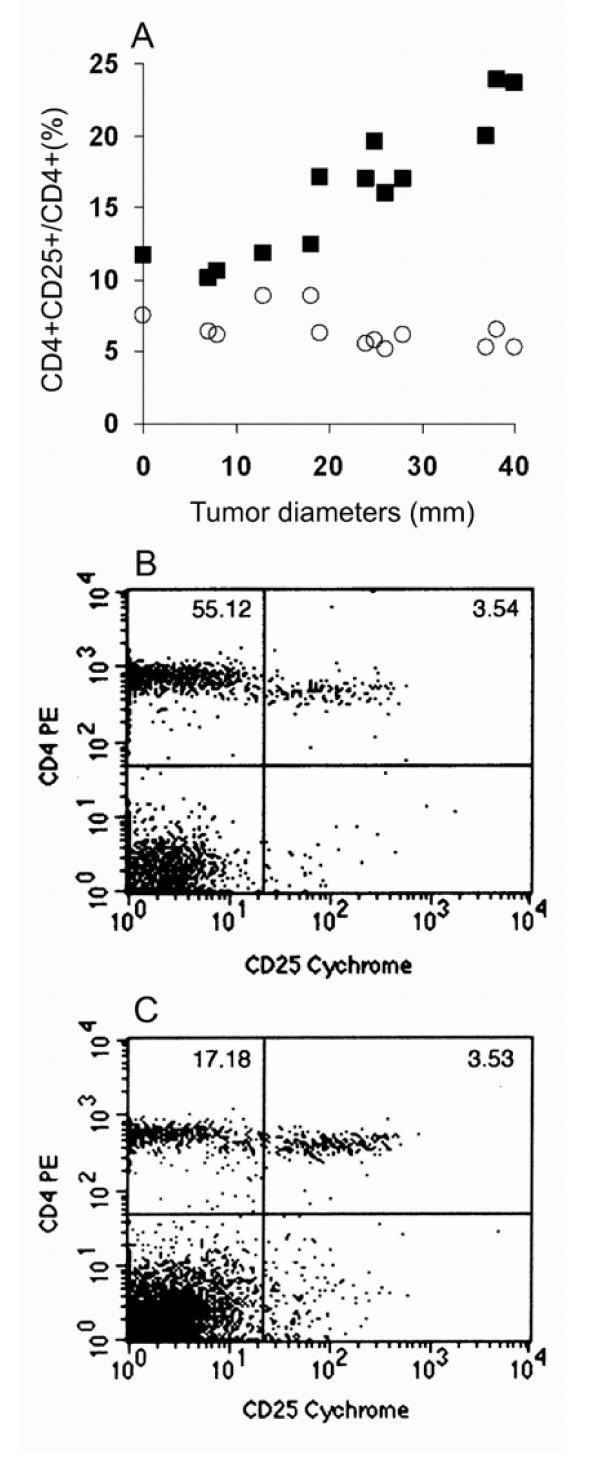
The relationship between tumor sizes and the CD4+CD25+/CD4+ proportions in peripheral blood or spleen lymphocytes in tumor bearing mice. (A) 1 × 10^5 ^to 1 × 10^7 ^C26 colon carcinoma cells were inoculated subcutaneously at right axilla of BALB/c mice (n = 12). 20 days later, after tumor sizes were measured, the mice were sacrificed and peripheral blood lymphocytes (○) and spleen (■) lymphocytes were stained with anti-mouse CD4 and CD25 antibodies. x-axis represents the diameters of tumors; y-axis represents the proportion of CD4+CD25+/CD4+. The absolute total lymphocyte counts were 9.85 ± 2.34 (× 10^9^/L) in peripheral blood and 2.37 ± 0.77 (× 10^8^) in spleen. The representative figures of CD4+CD25+ subset in peripheral blood (B) or spleen (C) lymphocytes from tumor bearing mice were also shown.

To investigate the possible changes of the proportion in tumor bearing mice, 1 × 10^5 ^to 1 × 10^7 ^live C26 colon carcinoma cells were inoculated subcutaneously at right axilla of BALB/c mice respectively (n = 12). 20 days later, tumor nodules were formed at different sizes from 7 to 40 mm in diameters. The mice were sacrificed and peripheral blood and spleen lymphocytes were prepared for double staining with anti-mouse CD4 and CD25 antibodies. In peripheral blood, we did not find an increase in CD4+CD25+/CD4+ in tumor bearing mice, compared with that in normal mice. Otherwise, an increased proportion of CD4+CD25+/CD4+ in spleen lymphocytes was observed in tumor bearing mice, moreover, the proportion increased in accordance with the increase in tumor sizes, as shown in Figure 2A. The representable double staining figure of peripheral blood or spleen lymphocytes from tumor bearing mice were shown in Figure 2B–C. Considering the short tumor bearing duration, we prolonged the observation to 50 to 60 days, the increase in the proportion was not yet observed in peripheral blood (data not shown).

### The changes of the percentages of CD4+CD25+, CD4+CD25- and total CD4+ cells in spleen lymphocytes from tumor bearing mice

**Figure 3:**
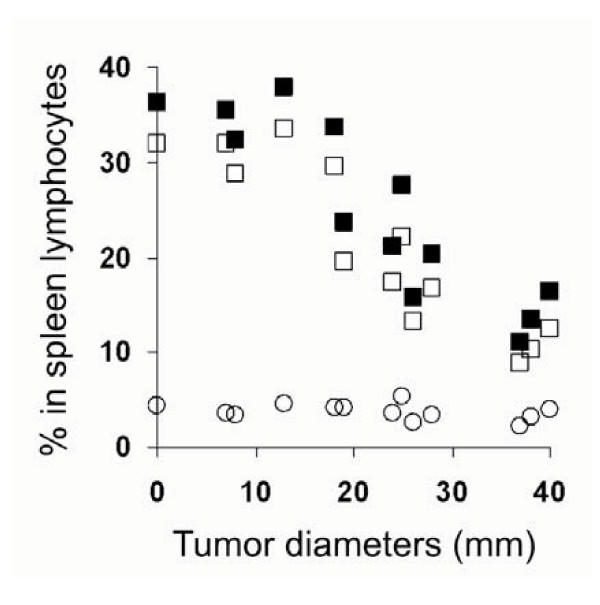
The relationship between tumor sizes and the percentages of total CD4+ (■), CD4+CD25- (□) or CD4+CD25+ (○) cells in spleen lymphocytes in tumor bearing mice. Samples were prepared and analyzed as in Figure 2, x-axis represents the diameter of tumors; y-axis represents the percentages in spleen lymphocytes.

The proportion of CD4+CD25+/CD4+ was determined by two factors: CD4+CD25+ in lymphocytes (numerator) and total CD4+ in lymphocytes (denominator). The increase of the proportion may be due to the increase of CD4+CD25+ subset or decrease of CD4+ subsets, or both. To investigate the possible reason that the proportion increased in spleen lymphocytes of tumor-bearing mice, we analyzed the CD4+CD25+ and total CD4+ cells in spleen lymphocytes. We found there was no obvious change in CD4+CD25+ in spleen lymphocytes from tumor bearing mice, otherwise, a decrease in total CD4+ lymphocytes was found with the increase of the tumor sizes, and the decrease was mainly due to the decrease of CD4+CD25- subset, as shown in Figure 3 and Figure 2C.

## Discussion

The identification of CD4+CD25+ as the phenotype of regulatory T lymphocytes is one of the highlights of recent immunological progress. These cells are proven to be involved in autoimmune diseases, transplantation tolerance and tumor immunity, etc [3]. The relationship between cancer and immune system has been studied and debated for a long time, now we know that immunodeficient or immunosuppressed humans or animals show greater incidences of cancer [22]; at the same time, immune function in cancer patients are often compromised by tumor itself or related treatment, and this often leads patients to disadvantageous situation. To restore the immune function in cancer patients is an important element in cancer treatment. The identification of CD4+CD25+ T_R _cells provided a new way to study relationship between tumor development and immune suppression. A higher proportion of CD4+CD25+ T_R _cells was found in peripheral blood of cancer patients and to be related to poor prognosis of the diseases [11,12]. Depletion of CD4+CD25+ T_R _cells using anti-CD25 mAb could promote anti-tumor immunity [4,15,16]. All these indicated that CD4+CD25+ T_R _cells maybe an attractive target to restore or improve immune function in cancer treatment.

In our animal experiments of antitumor immunotherapy, we did not find an increase of CD4+CD25+/CD4+ in peripheral blood in tumor bearing BALB/c mice, this is not in accordance with the results in cancer patients reported previously [11]. To find a way to evaluate the CD4+CD25+/CD4+ in antitumor immunotherapy targeting CD4+CD25+ T_R _cells, we analyzed the proportion in peripheral blood and spleen lymphocytes in normal or C26 colon-carcinoma-bearing mice by flow cytometry. In present study, the proportion of CD4+CD25+/CD4+ in peripheral blood of normal mice was about 6.19%, which was compatible with the results reported previously (5–10%). But in spleen lymphocytes from normal mice, we found a higher proportion of CD4+CD25+/CD4+ (around 10%), and the higher proportion is due to a lower level of total CD4+ lymphocytes in spleen, compared with that in peripheral blood, whereas the percentages of the CD4+CD25+ cells are similar.

In C26-colon-carcinoma bearing BALB/c mice, we found an increase of CD4+CD25+/CD4+ in spleen but not in peripheral blood, furthermore, the proportion in spleen lymphocytes increased with the increase of tumor sizes. The phenomenon that the increase of the proportion in spleen separates with that in peripheral blood may be due to: 1). Spleen is a professional immune organ, which maybe more sensitive to the changes of immune situation than peripheral blood; 2). In this study, what we used is artificial tumor model, not spontaneous tumor model, and the tumor grew so quickly to cause mice moribund or dead that the increase of the proportion did not appear in peripheral blood. To observe the increase of the proportion in peripheral blood of tumor bearing mice may need a longer observation duration, or had better use spontaneous tumor models. In our experiments, we found the increase of CD4+CD25+/CD4+ is due to the decrease of CD4+ in lymphocytes, which is the result of decreased CD4+CD25- subset in lymphocytes. Our results support the observations reported by Sasada [12], in which the relative increase in the proportion of CD4+CD25+ T cells in patients with gastrointestinal malignancies are due to a selective reduction in the number of CD4+CD25- T cells. A possible explanation for this is that CD4+CD25- subset is more sensitive to clonal deletion or apoptosis than CD4+CD25+ T cells [12,23,24]. Furthermore, it is possible that some factors, such as tumor-derived antigens or molecules, can induce apoptosis selectively in the CD4+CD25- subset but not in the CD4+CD25+ subset [12].

The relationship between cancer and immune system has been debated for a long time. Our results provided direct evidence that the tumor might compromise the immune function, since our tumor model was established on BALB/c mice with normal immune function. It is known that tumor cells secrete immunosuppressive cytokines such as IL-10 and TGF-β [25-27], and the cytokines may induce CD4+CD25- lymphocytes to convert to CD4+CD25+ T_R _cells [28,29]. These all support the theory that tumor may compromise the immune function.

## Conclusions

In normal BALB/c mice, CD4+CD25+/CD4+ proportion in spleen lymphocytes is higher than that in peripheral blood lymphocytes. In C26-colon-carcinoma bearing mice, no difference was found in the proportion in peripheral blood lymphocytes compared with normal mice; Otherwise, the proportion in spleen lymphocytes obviously increased, moreover, the proportion increased in accordance with the increase of tumor sizes. The increase of the proportion is due to the decrease of total CD4+ in lymphocytes, which is resulted from decreased CD4+CD25- subset in lymphocytes. Our observation suggest the CD4+CD25+/CD4+ proportion in spleen lymphocytes might be a sensitive index to evaluate the T_R _in tumor mouse models rather than that in peripheral blood lymphocytes, and our results provide some information on strategies of antitumor immunotherapy targeting CD4+CD25+ regulatory T lymphocytes.
